# Autosomal InDel polymorphisms for population genetic structure and differentiation analysis of Chinese Kazak ethnic group

**DOI:** 10.18632/oncotarget.17838

**Published:** 2017-05-12

**Authors:** Tingting Kong, Yahao Chen, Yuxin Guo, Yuanyuan Wei, Xiaoye Jin, Tong Xie, Yuling Mu, Qian Dong, Shaoqing Wen, Boyan Zhou, Li Zhang, Chunmei Shen, Bofeng Zhu

**Affiliations:** ^1^ Key Laboratory of Shaanxi Province for Craniofacial Precision Medicine Research, College of Stomatology, Xi’an Jiaotong University, Xi’an, P. R. China; ^2^ Clinical Research Center of Shaanxi Province for Dental and Maxillofacial Diseases, College of Stomatology, Xi’an Jiaotong University, Xi’an, P. R. China; ^3^ Department of Forensic Genetics, School of Forensic Medicine, Southern Medical University, Guangzhou, P. R. China; ^4^ Laboratory Medicine Center, The First Affiliated Hospital of Xinjiang Medical University, Urumqi, P. R. China; ^5^ College of Medicine & Forensics, Xi’an Jiaotong University Health Science Center, Xi’an, P. R. China; ^6^ State Key Laboratory of Genetic Engineering and MOE Key Laboratory of Contemporary Anthropology, School of Life Sciences and Institutes of Biomedical Sciences, Fudan University, Shanghai, P. R. China; ^7^ State Key Laboratory of Genetic Engineering and Institute of Biostatistics, School of Life Sciences, Fudan University, Shanghai, P. R. China; ^8^ Department of Gastroenterology, Second Affiliated Hospital of Xi’an Jiaotong University, Xi’an, P. R. China; ^9^ Institute of Brain and Behavioral Sciences, College of Life Sciences, Shaanxi Normal University, Xi’an, P. R. China

**Keywords:** Kazak group, InDel, population genetics, interpopulation differentiation, phylogenetic tree

## Abstract

In the present study, we assessed the genetic diversities of the Chinese Kazak ethnic group on the basis of 30 well-chosen autosomal insertion and deletion loci and explored the genetic relationships between Kazak and 23 reference groups. We detected the level of the expected heterozygosity ranging from 0.3605 at HLD39 locus to 0.5000 at HLD136 locus and the observed heterozygosity ranging from 0.3548 at HLD39 locus to 0.5283 at HLD136 locus. The combined power of discrimination and the combined power of exclusion for all 30 loci in the studied Kazak group were 0.999999999999128 and 0.9945, respectively. The dataset generated in this study indicated the panel of 30 InDels was highly efficient in forensic individual identifcation but may not have enough power in paternity cases. The results of the interpopulation differentiations, PCA plots, phylogenetic trees and STRUCTURE analyses showed a close genetic affiliation between the Kazak and Uigur group.

## INTRODUCTION

In the last few years, a novel polymorphic marker, insertion and deletion polymorphisms (InDels), gained increased concern and attention in the field of medical and forensic genetics. InDels as biallelic markers combine desirable characteristics of both single nucleotide polymorphisms (SNPs) and short tandem repeats (STRs), becoming promising genetic markers for forensic purposes. InDels could be readily analyzed by capillary electrophoresis which is relatively common in the forensic DNA laboratories [1]. Moreover, InDels have small amplicons and relatively low mutation rates, which makes them more applicable for degraded or ancient DNA analyses [2, 3]. Besides the purpose of forensic caseworks, ancestry informative InDels can be good candidates for biogeographic ancestry analyses since the allele frequencies of InDels are significantly different between different groups or populations [4].

The Kazak national minority whose population exceeds a million mainly lives in the Ili Kazak Autonomous Prefecture, Barkol Kazak Autonomous County and Mori Kazak Autonomous County in the Xinjiang Uygur Autonomous Region, China. Some are located in Qinghai and Gansu Provinces (http://en.people.cn/102759/102835/7562907.html). To date, one commercial kit, Qiagen Investigator DIPplex reagent (Qiagen, Hilden, Germany), has been applied in multiplex amplification of 30 autosomal InDels. Previous population studies have been done and published using the kit [5–8]. In order to further clarify the genetic background and origin of the Kazak ethnic minority, we collected bloodstain samples from Kazak group in Xinjiang Uygur Autonomous Region and obtained population data using the kit mentioned above. Then we calculated the statistical parameters of the 30 autosomal InDel loci and evaluated the population genetic differentiations between Kazak and 23 previously published populations.

## RESULTS AND DISCUSSIONS

### Forensic statistical parameter analysis

**Figure 1:**
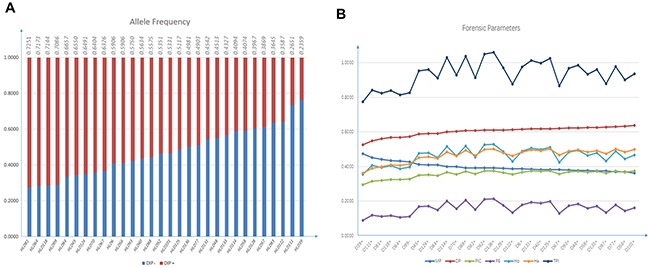
Plots graphs of allele frequency **(A)** and forensic parameters **(B)** of Kazak group on 30 InDel loci. The frequencies of insertion alleles were presented on the top of (A).

Allele frequencies and forensic efficiency parameters of 30 InDels in Kazak group were shown in Figure 1 and Supplementary Table 1. Having applied the Bonferroni correction, Hardy-Weinberg equilibrium (HWE) test showed no significant deviation from the expected value (*p*>0.0017), with the lowest *p* value at HLD97 locus (0.0455). We observed the expected heterozygosity (He) ranging from 0.3605 (HLD39) to 0.5000 (HLD136) and the observed heterozygosity (Ho) ranging from 0.3548 (HLD39) to 0.5283 (HLD136). The match probability (MP), the typical paternity index (TPI) and the polymorphic information content (PIC) were in the range of 0.3619 to 0.4736, 0.7749 to 1.0599 and 0.2955 to 0.3750, with a mean value of 0.3971, 0.9344 and 0.3555, respectively. The power of discrimination (DP) ranged from 0.5264 (HLD39) to 0.6381 (HLD101). The highest value of the power of exclusion (PE) was 0.2135 observed at HLD136 locus, while the lowest value was 0.0887 at HLD39 locus. The combined power of discrimination (CDP) and the combined power of exclusion (CPE) for all 30 loci in Kazak group were 0.999999999999128 and 0.9945, respectively. The high CDP demonstrated the sufficient potential of the 30 InDels in forensic individual identification. However, compared with the previous study concerning 21 STR loci in Kazak group [9], the CPE value was relatively low, which suggested that the panel of 30 InDels could just be treated as a supplement for STR loci in kinship analyses.

### Linkage disequilibrium analysis

Linkage disequilibrium test between 30 InDels was carried out using the SNPAnalyzer program. As shown in Supplementary Figure 1, no crimson color coated by thick black curve existing in the graph and there was no significant LD observed between pairwise InDels with the values of *r*^2^ less than 0.1(data not shown). Thus, these genetic markers could be regarded as relatively independent in the subsequent statistical analyses.

### Interpopulation differentiations

Interpopulation differentiations were compared using the analyses of molecular variance (AMOVA) method. As shown in Supplementary Table 2, we calculated pairwise *p* values between the studied Kazak group and 23 previously published populations including Kazak1 [7], Uigur [7], Yi [10], Xibe [11], Tujia [12], South Korean [13], She [14], two Tibetan groups [15], three Han populations [7, 14, 16] in different regions, six Mexican groups [17], four European groups and Uruguayan group [18–21] (The geographical locations of the studied Kazak and other reference populations were shown in Supplementary Figure 2) based on allele frequencies of 30 InDel loci. The least differences were observed between the studied Kazak group and Kazak1, Uigur groups with significant differences at 1, 6 loci, respectively. While the most significant differences were found between Kazak group and six Mexican groups at 21-26 loci. Among the 30 loci, the HLD111 and HLD81 loci showed the highest population genetic differentiations with significant differences between Kazak group and 21 other compared populations, and the HLD77, HLD93, HLD101 and HLD136 loci had the lowest ethnic diversities with just 7 pair-wise populations. The present results showed that there were significant differences in allele frequency distributions of some InDel loci among different ethnic groups. Hence, study of more InDel allelic distributions in more populations may be required for the forensic application researches.

**Figure 2:**
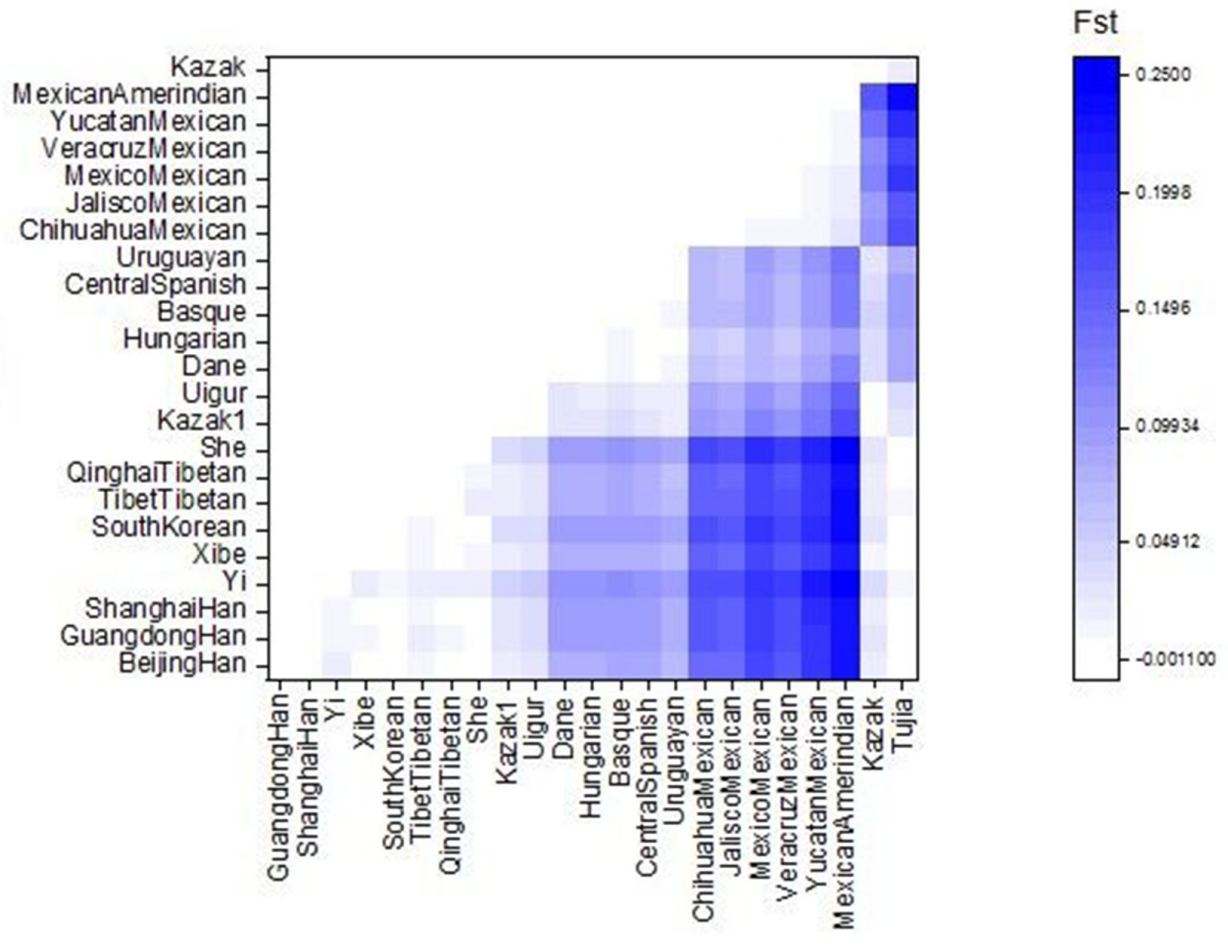
Plots of pairwise *Fst* of Kazak and other 23 reference populations

As shown in Figure 2, a heat map of pairwise *Fst* of Kazak and other referenced population was carried out by *R* statistical software [22]. A shade of blue color in the heat map represents the genetic distances of pairwise populations. The darker color stands for the bigger *Fst* value and the farther genetic relationship. On the contrary, the lighter color stands for the samller *Fst* value and the closer genetic relationship. It is obvious that close genetic relationships could be observed again between the studied Kazak group and Kazak1, Uigur groups, which would be displayed directly as labels of the lighter color for their pairwise *Fst* values.

### PCA and STRUCTURE analysis

**Figure 3:**
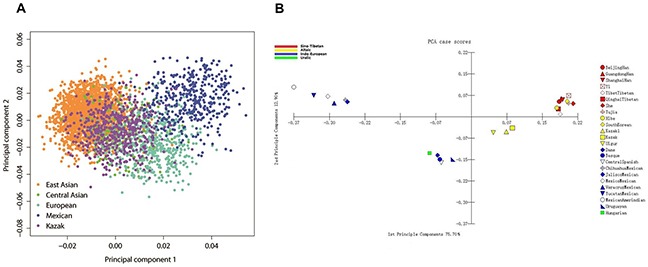
PCA analyses based on 30 InDel loci **(A)** Kazak, East Asian, Central Asian, Mexican and European populations were analyzed at individual level. (Here, European populations included Basque, Central Spanish, Dane, Hungarian as well as Uruguayan populations.) **(B)** The studied Kazak group and other 23 published populations.

The principal component analyses (PCA) were conducted between Kazak group and other 23 populations utilizing the softwares of EIGENSOFT 6.0.1 [23] and MATLAB 2007a, respectively. As shown in Figure 3a, 24 populations were divided into four main regions in good accordance with their geographic distributions, that is East Asians (Han, Yi, Tujia, Tibetan, South Korean, Xibe and She); Europeans (Basque, Central Spanish, Dane, Uruguayan and Hungarian); Mexicans and Central Asians (Kazak, Kazak1 and Uigur). The Kazak, Kazak1 and Uigur groups as expected scattered in the middle of East Asians and Europeans, which revealed the close genetic relationship between Kazak and Uigur groups. In another PCA plot (shown in Figure 3b), the first two principal components accounted for 88.60% of the total variance. Four distinct areas were observed, and the Kazak group similarly clustered to the Kazak1 and Uigur groups.

**Figure 4:**
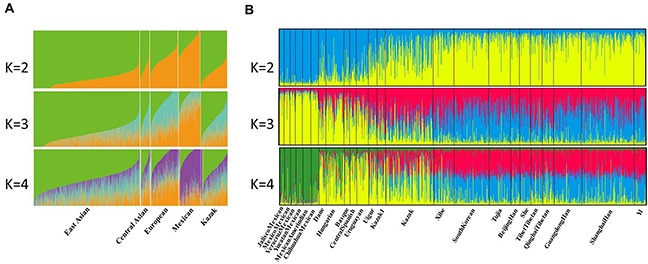
**(A)** Admixture analysis of 24 populations at *K*=2, 3, 4 using ADMIXTURE program. **(B)** STRUCTURE analysis conducted with STRUCTURE program v2.2.

We then carried out the STRUCTURE analysis of the 24 populations with ADMIXTURE v1.23 software [24] which is a useful tool to infer individual genetic ancestry coefficients by conditioning the value of *K* (the number of hypothetical ancestral populations) and thus analyze population structure. Although the 30 InDel loci are not ideal ancestry informative markers and have limited differentiation power, they were still efficient to distinguish ancestries of the studied Kazak and other populations to some extent. As shown in Figure 4a, the ancestry components of Kazak group were similar to that of Central Asians (Uigur and Kazak1 groups) with different *K* value. Of course, it is still need more effective ancestry informative markers to identify and estimate ancestry components of admixtures better in the later studies. Moreover, we performed the population structure analysis again with the STRUCTURE program v2.2, which is given in Figure 4b. At *K*=2, the Asians and Europeans were almost entirely filled with yellow and blue component, respectively. Meanwhile, the Kazak, Kazak1 and Uigur groups represented a mixture of blue and yellow components. Uigur and Kazak groups could be better separated from Europeans and East Asians at *K*=3, which was in accordance with the result of output posterior probabilities that *K*=3 was the most appropriate and suitable configuration (shown in Supplementary Figure 3).

### Genetic distances and phylogenetic analysis

Genetic distance (*D_A_* distance) reveals the genetic divergence between different populations. Populations with similar allelic distributions have small genetic distances. In order to estimate the genetic distances between the Kazak and 23 reference populations, we calculated *D_A_* values on the basis of 30 InDels. As presented in Table 1, the smallest distance was showed between the Kazak and Kazak1 group (*D_A_*=0.0007), followed by Uigur group with *D_A_*=0.0016. And the largest genetic distance was found with Mexican Amerindian group (*D_A_*=0.0526), which were consistent with the *Fst* and PCA results mentioned above.

**Table 1:** The values of *D*_*A*_ of pairwise populations among Chinese Kazak group and referenced populations

Populations	BeijingHan	Guangdong Han	ShanghaiHan	Yi	Xibe	SouthKorean	TibetTibetan	QinghaiTibetan	She	Kazak1	Uigur	Dane	Hungarian	Basque	CentralSpanish	Uruguayan	ChihuahuaMexican	JaliscoMexican	MexicoMexican	VeracruzMexican	YucatanMexican	MexicanAmerindian	Kazak
Guangdong-Han	0.0019																						
Shanghai-Han	0.0011	0.0006																					
Yi	0.0054	0.0038	0.0040																				
Xibe	0.0022	0.0023	0.0015	0.0052																			
South-Korean	0.0024	0.0017	0.0008	0.0042	0.0016																		
Tibet-Tibetan	0.0029	0.0050	0.0036	0.0066	0.0034	0.0037																	
Qinghai-Tibetan	0.0022	0.0034	0.0021	0.0056	0.0019	0.0023	0.0012																
She	0.0023	0.0015	0.0019	0.0051	0.0032	0.0028	0.0059	0.0041															
Kazak1	0.0083	0.0100	0.0096	0.0133	0.0068	0.0115	0.0072	0.0075	0.0112														
Uigur	0.0100	0.0118	0.0114	0.0163	0.0092	0.0135	0.0091	0.0089	0.0133	0.0013													
Dane	0.0251	0.0265	0.0264	0.0315	0.0227	0.0288	0.0242	0.0235	0.0275	0.0093	0.0083												
Hungarian	0.0255	0.0275	0.0271	0.0325	0.0231	0.0295	0.0237	0.0232	0.0289	0.0084	0.0068	0.0026											
Basque	0.0270	0.0268	0.0270	0.0328	0.0236	0.0287	0.0261	0.0256	0.0288	0.0111	0.0096	0.0048	0.0045										
Central-Spanish	0.0262	0.0269	0.0268	0.0323	0.0226	0.0288	0.0236	0.0235	0.0285	0.0085	0.0069	0.0030	0.0022	0.0033									
Uruguayan	0.0230	0.0244	0.0240	0.0286	0.0203	0.0258	0.0207	0.0201	0.0255	0.0067	0.0057	0.0039	0.0021	0.0043	0.0023								
Chihuahua-Mexican	0.0445	0.0471	0.0465	0.0522	0.0441	0.0500	0.0460	0.0442	0.0525	0.0278	0.0241	0.0183	0.0156	0.0212	0.0200	0.0207							
Jalisco-Mexican	0.0437	0.0450	0.0448	0.0512	0.0420	0.0484	0.0452	0.0430	0.0502	0.0260	0.0225	0.0170	0.0137	0.0197	0.0181	0.0189	0.0017						
Mexico-Mexican	0.0534	0.0548	0.0544	0.0602	0.0517	0.0576	0.0545	0.0528	0.0606	0.0345	0.0313	0.0222	0.0210	0.0256	0.0258	0.0274	0.0048	0.0041					
Veracruz-Mexican	0.0498	0.0500	0.0502	0.0566	0.0473	0.0535	0.0501	0.0483	0.0555	0.0301	0.0263	0.0192	0.0160	0.0209	0.0197	0.0223	0.0040	0.0024	0.0030				
Yucatan-Mexican	0.0588	0.0599	0.0598	0.0675	0.0569	0.0633	0.0613	0.0587	0.0655	0.0391	0.0351	0.0267	0.0230	0.0277	0.0279	0.0300	0.0048	0.0046	0.0041	0.0031			
Mexicann-Amerindian	0.0718	0.0724	0.0732	0.0798	0.0698	0.0770	0.0752	0.0718	0.0796	0.0519	0.0477	0.0365	0.0317	0.0389	0.0393	0.0427	0.0093	0.0090	0.0087	0.0064	0.0053		
Kazak	0.0064	0.0079	0.0073	0.0115	0.0048	0.0087	0.0060	0.0057	0.0093	0.0007	0.0016	0.0100	0.0092	0.0115	0.0092	0.0073	0.0285	0.0267	0.0355	0.0309	0.0404	0.0526	
Tujia	0.0013	0.0007	0.0004	0.0040	0.0015	0.0009	0.0035	0.0023	0.0019	0.0104	0.0125	0.0272	0.0281	0.0281	0.0277	0.0250	0.0495	0.0480	0.0576	0.0534	0.0632	0.0764	0.0079

**Figure 5:**
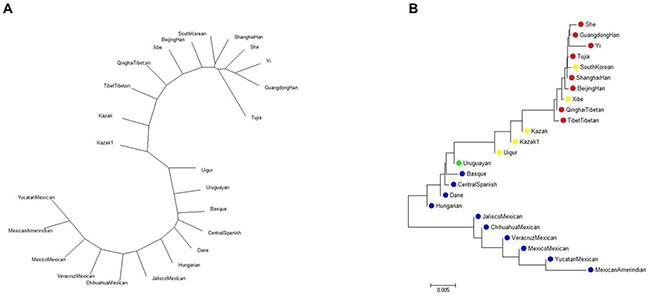
Phylogenetic trees constructed to analyze phylogenetic relationships between Kazak group and 23 reference populations **(A)** On the basis of allele frequencies of 30 InDels conducted by PHYLIP software. **(B)** With the method of MAGA software (version5.0) based on *D_A_* values calculated by DISPAN program.

We further conducted phylogenetic reconstruction on the basis of two different methods. An unrooted tree was constructed by the PHYLIP software (version3.6) based on the allele frequencies of all InDel loci, revealing the genetic relationships between studied kazak group and other compared populations. As shown in the Figure 5a, the branch on the top side contained ten East Asian groups, whereas the lower one consisted of Uruguayan group, four European populations and six Mexican populations. The Kazak, Kazak1 and Uigur groups were in the middle of the above two branches. Based on the *D_A_* distances, a phylogenetic tree reconstructed by MAGA software using neighbor joining (N-J) method was in the Figure 5b, and two main clusters could be seen from the dendrogram. The first cluster was composed of the East Asian groups, Central Asian groups(Kazak, Kazak1 and Uigur groups), Uruguayan group, as well as European groups, while the second one consisted of six Mexican groups. The Kazak group firstly tended to cluster together with Kazak1 and Uigur groups, and then with other groups, which indicated close relationships between the studied Kazak and Kazak1, Uigur groups. Yuan et al. represented a N-J tree with regard to 21 autosomal STR loci and also found Kazak was closely related to Uigur [10]. The dendrogram based on 17 Y-chromosomal STRs indicated the close relationship between Kazak and Uigur as well [25]. The same results were also obtained from the previous HLA and mtDNA studies [26, 27]. The clustering results of phylogenetic trees were in good accordance with the above results of the inter-population differentiations, PCA plots, genetic distances and STRUCTURE analyses. In Chinese history, Uigurs belonged to a branch of Turkic people, while the Kazaks were formed as a result of the long-term development of the Turkic, Wusun, Khitan and Mongolian people. Furthermore, Kazaks and Uigurs were the main populations in the Silk Road of ancient China and they shared the common religious belief and culture, which indicated the two groups located in Central Asia have had a close geographic connection since ancient times. Therefore, the gene flow might exist and bring about the close affiliation between Kazak and Uigur groups.

## MATERIALS AND METHODS

### Samples collection and DNA isolation

Bloodstain samples were collected from 513 un- related healthy individuals (200 males and 313 females) from Kazak group residing in Xinjiang Uygur Autonomous Region, China. Written informed consents were acquired from all participants involved in this study. The research was carried out according to the human and ethical research principles of Xi’an Jiaotong University Health Science Center, China and approved by the ethics committee of Xi’an Jiaotong University Health Science Center. In the process of collecting samples, it must be ensured that any two individuals have no any blood relationship within at least three generations. Human genomic DNA was isolated from bloodstain samples utilizing the method of Chelex-100 [28].

### PCR amplification and InDels genotyping

A multiple PCR amplification with fluorescent of autosomal 30 InDels was conducted with Investigator DIPplex reagent (Qiagen, Hilden, Germany) in a single multiplex reaction on GeneAmp PCR System 9700 thermal cycler (Applied Biosystems, Foster City, CA, USA) following manufacturer's instructions. InDel genotyping was performed with capillary electrophoresis on ABI 3500 Genetic Analyzer (Applied Biosystems, Foster City, CA, USA) and analyzed by GeneMapper v3.2 software (Applied Biosystems, Foster City, CA, USA).

### Statistical analysis

Allele frequency distributions and forensic statis-tical parameters including HWE, MP, Ho, PE, DP, PIC and TPI of 30 InDel loci were computed with the modified Powerstat (version1.2) spreadsheet (Promega, Madison, WI, USA). And the He values were calculated as described previously [29]. The locus-by-locus *p* values were estimated by Arlequin software (version3.0) using the AMOVA method. The heat map of pairwise *Fst* was carried out in *R* statistical software v3.0.2. The SNPAnalyzer (version2.0 Istech, South Korea) was used to test LD for all pair-wise InDels. Two PCA plots were carried out in EIGENSOFT v6.0.1 software and MATLAB 2007a (MathWorks Inc., USA), respectively. Population genetic structure analyses were performed with the ADMIXTURE v1.23 and STRUCTURE v2.2 programs. Two phylogenic trees were conducted by PHYLIP (version3.6) software via allele frequencies of 30 InDels, as well as MAGA software (version5.0) based on *D_A_* values, respectively.

## CONCLUSION

In this study, we obtained the allele frequencies and forensic parameters of the autosomal 30 InDels for the research of population genetics and forensic sciences. And we found the panel was highly efficient in forensic individual identification but could only be used as supplementary markers for STR loci in paternity cases. The results of the interpopulation differentiations, PCA plots, phylogenic trees and STRUCTURE analyses indicated a close genetic relationship between Kazak and Uigur groups. In order to better understand the origin and genetic background of Kazak group, further study should be conducted in later research.

## SUPPLEMENTARY MATERIALS FIGURES AND TABLES




