# Supplementary material for: Autosomal InDel polymorphisms for population genetic structure and differentiation analysis of Chinese Kazak ethnic group
Source: Oncotarget. 2017 May 12;8(34):56651–8. doi: 10.18632/oncotarget.17838 (PMC5593590; doi:10.18632/oncotarget.17838)
Supplement: Supplementary file 1 [file oncotarget-08-56651-s001.pdf]

Autosomal InDel polymorphisms for population genetic structure and differentiation analysis of Chinese Kazak ethnic group

SUPPLEMENTARY MATERIALS

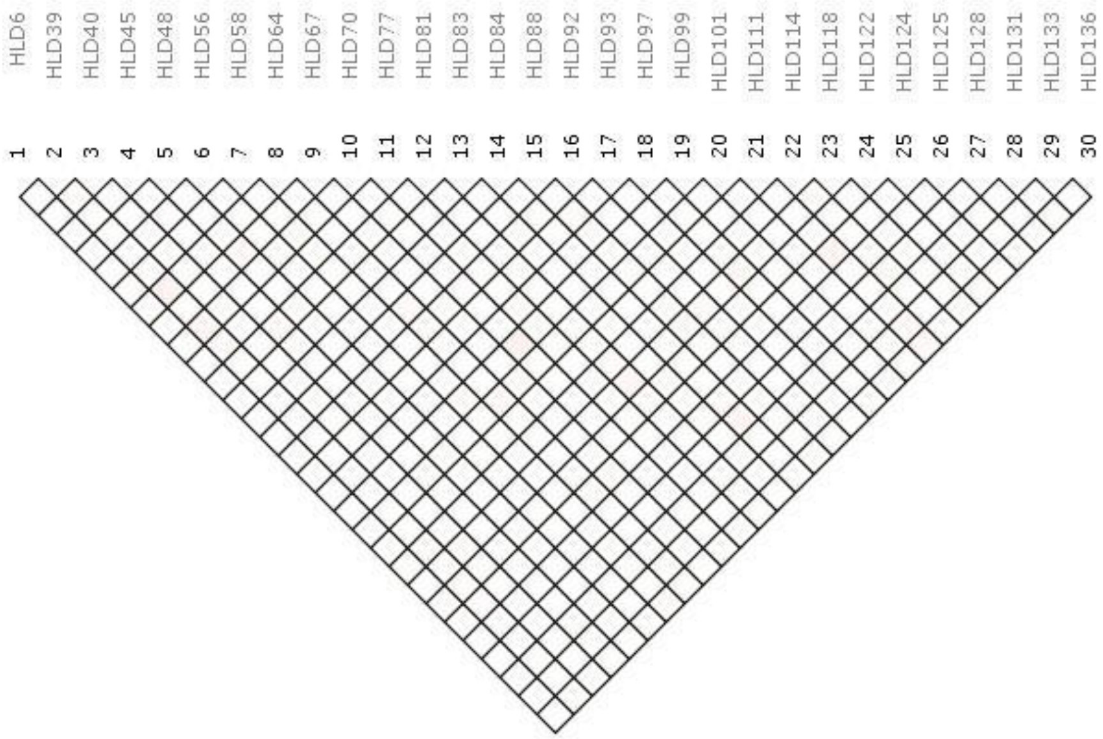

Supplementary Figure 1: The LD analysis schema between the 30 InDel loci using the SNPAnalyzer 2.0 program. There was no crimson color coated by thick black curve existing in the graph.

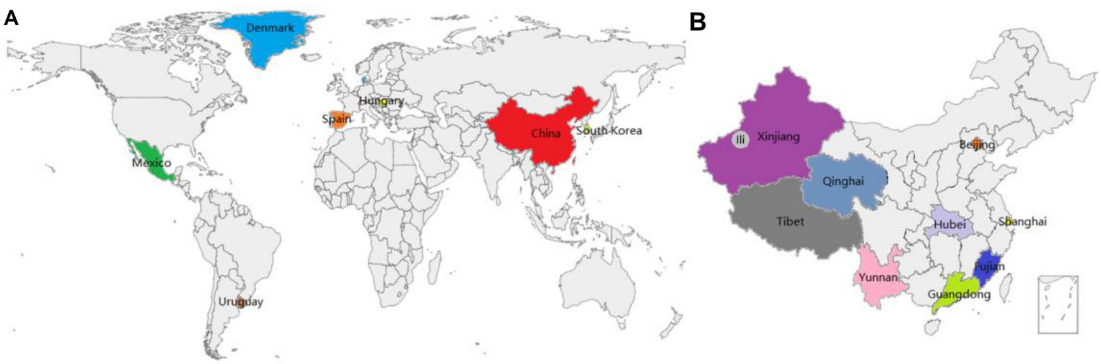

Supplementary Figure 2: Geographic location of all 24 populations in this study. (A) The world map showing the geographic location of all populations, including the South Korean group from South Korea (Seoul, Daejeon, Busan, Gwangju, and Daegu), Dane group from Denmark, Hungarian group from Hungary, Basque group from Basque, Central Spanish group from Spain (Madrid), Uruguayan group from Uruguay (Center, South, Littoral, and Northeast), 6 Amerindian groups from Mexico, 12 Chinese groups in China. **(B)** The Chinese map showing the geographic location of Kazak group and 11 Chinese populations, including the Kazak1 group from Ili, Uyghur group from Urumqi, Yi group from Yunnan province, Xibe group from Ili, Tujia group from Hubei province, She group from Fujian province, TibetTibetan group from Tibet, QinghaiTibetan group from Qinghai provincINe, 3 Han groups from Beijing, Shanghai and Guangdong province, respectively. Kazak group in this study from Xinjiang Uygur Autonomous Region.

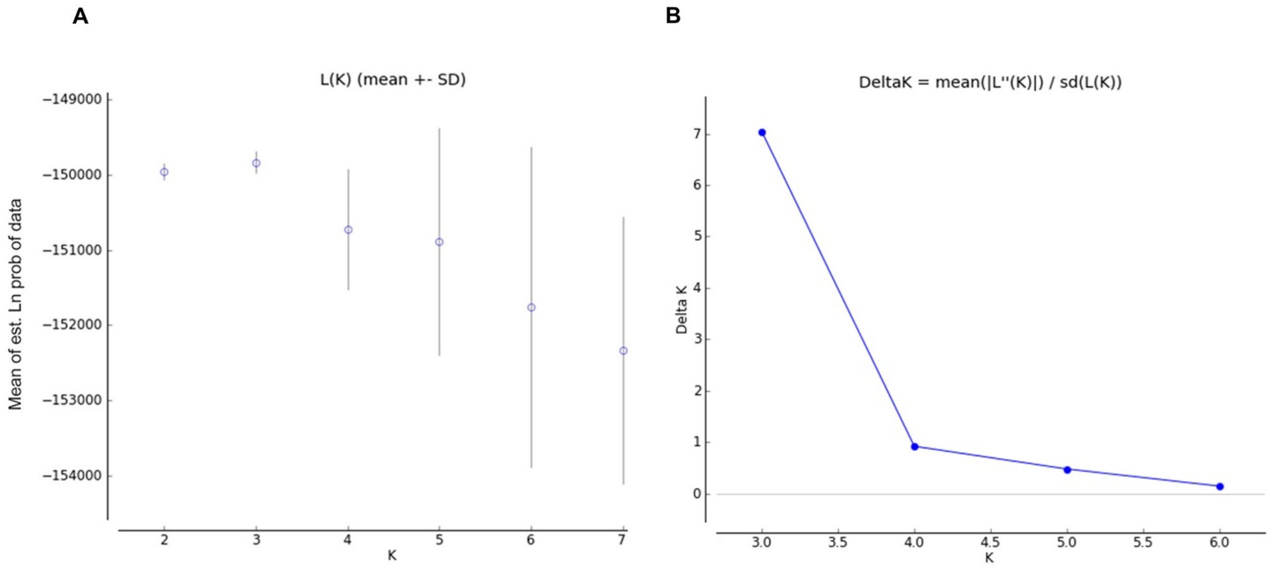

Supplementary Figure 3: Diagram form of the estimated probability of data. (A) Plot of mean likelihood L(K) and variance at *K*=2-7. We could find a plateau at *K*=3, 4. **(B)** The maximum of delta *K* value was calculated at *K*=3.

Supplementary Table 1: Allele frequency distribution and forensic statistical parameters of the 30 InDel loci in Chinese Kazak ethnic group (n=513)

HLD	rs#	DIP+	DIP-	Ho	He	*p*	TPI	PIC	PE	DP	MP
6	1610905	0.5906	0.4094	0.4873	0.4836	0.8815	0.9753	0.3666	0.1767	0.6147	0.3853
39	17878444	0.2359	0.7641	0.3548	0.3605	0.7756	0.7749	0.2955	0.0887	0.5264	0.4736
40	2307956	0.5634	0.4366	0.4990	0.4920	0.7659	0.9981	0.3710	0.1866	0.6175	0.3825
45	2307959	0.6550	0.3450	0.4756	0.4520	0.2906	0.9535	0.3498	0.1671	0.5883	0.4117
48	28369942	0.4513	0.5487	0.4932	0.4953	0.9078	0.9865	0.3726	0.1816	0.6236	0.3764
56	2308292	0.5906	0.4094	0.4444	0.4836	0.0727	0.9000	0.3666	0.1433	0.6317	0.3683
58	1610937	0.4074	0.5926	0.4639	0.4829	0.3796	0.9327	0.3663	0.1578	0.6239	0.3761
64	1610935	0.7173	0.2827	0.3860	0.4055	0.3575	0.8143	0.3233	0.1056	0.5680	0.4320
67	1305056	0.6326	0.3674	0.4230	0.4649	0.0547	0.8666	0.3568	0.1285	0.6195	0.3805
70	2307652	0.6404	0.3596	0.4620	0.4606	0.9661	0.9293	0.3545	0.1564	0.6024	0.3976
77	1611048	0.4903	0.5097	0.4893	0.4998	0.6177	0.9790	0.3749	0.1783	0.6300	0.3700
81	17879936	0.7251	0.2749	0.3938	0.3986	0.8083	0.8248	0.3192	0.1102	0.5598	0.4402
83	2308072	0.3645	0.6355	0.4522	0.4633	0.6014	0.9128	0.3560	0.1490	0.6087	0.3913
84	3081400	0.6657	0.3343	0.4503	0.4451	0.8281	0.9096	0.3460	0.1476	0.5912	0.4088
88	8190570	0.5575	0.4425	0.5185	0.4934	0.2641	1.0385	0.3717	0.2042	0.6086	0.3914
92	17174476	0.5351	0.4649	0.5244	0.4975	0.2327	1.0512	0.3738	0.2097	0.6095	0.3905
93	2307570	0.5750	0.4250	0.4834	0.4887	0.7934	0.9679	0.3693	0.1734	0.6216	0.3784
97	17238892	0.3869	0.6131	0.4308	0.4744	0.0455	0.8784	0.3619	0.1338	0.6269	0.3731
99	2308163	0.7086	0.2914	0.3957	0.4130	0.4160	0.8274	0.3277	0.1113	0.5738	0.4262
101	2307433	0.5331	0.4669	0.4659	0.4978	0.1422	0.9361	0.3739	0.1594	0.6381	0.3619
111	1305047	0.2651	0.7349	0.4055	0.3897	0.4738	0.8410	0.3137	0.1173	0.5485	0.4515
114	2307581	0.4094	0.5906	0.5146	0.4836	0.1658	1.0301	0.3666	0.2006	0.6009	0.3991
118	16438	0.7144	0.2856	0.4035	0.4080	0.8202	0.8382	0.3248	0.1161	0.5673	0.4327
122	8178524	0.3587	0.6413	0.4288	0.4601	0.1503	0.8754	0.3542	0.1325	0.6130	0.3870
124	6481	0.6491	0.3509	0.4795	0.4555	0.2839	0.9607	0.3518	0.1702	0.5901	0.4099
125	16388	0.5117	0.4883	0.5117	0.4997	0.6026	1.0240	0.3749	0.1980	0.6187	0.3813
128	2307924	0.3967	0.6033	0.4854	0.4787	0.7765	0.9716	0.3641	0.1750	0.6106	0.3894
131	1611001	0.4542	0.5458	0.5068	0.4958	0.6332	1.0138	0.3729	0.1935	0.6173	0.3827
133	2067235	0.4327	0.5673	0.4795	0.4910	0.5898	0.9607	0.3704	0.1702	0.6256	0.3744
136	16363	0.4981	0.5019	0.5283	0.5000	0.2082	1.0599	0.3750	0.2135	0.6097	0.3903

HLD, human locus deletion/insertion polymorphism; DIP-, frequency of short allele; DIP+, frequency of long allele; Ho, observed heterozygosity; He, expected heterozygosity; *p*, *p* value for Hardy-Weinberg equilibrium; TPI, typical paternity index; PIC, polymorphic information content; PE, power of exclusion; DP, power of discrimination; MP, match probability.

Supplementary Table 2: The *P* values of pairwise InDel loci between Chinese Kazak group and referenced populations at 30 InDel loci

See Supplementary File 1
