# Supplementary material for: Autosomal InDel polymorphisms for population genetic structure and differentiation analysis of Chinese Kazak ethnic group
Source: Oncotarget. 2017 May 12;8(34):56651–8. doi: 10.18632/oncotarget.17838 (PMC5593590; doi:10.18632/oncotarget.17838)
Supplement: Supplementary file 2 [file oncotarget-08-56651-s002.doc]

**Supplementary Table 2: The *p* values of pairwise InDel loci between Chinese Kazak group and referenced populations at 30 InDel loci**

| Loci | Beijing  Han | Guangdong  Han | Shanghai  Han | Yi | Xibe | South  Korean | Tibet  Tibetan | Qinghai  Tibetan | She | Kazak1 | Uigur | Dane | Hungarian | Basque |
| --- | --- | --- | --- | --- | --- | --- | --- | --- | --- | --- | --- | --- | --- | --- |
| D6 | **0.0000** | **0.0000** | **0.0000** | **0.0000** | 0.1075 | **0.0000** | **0.0166** | 0.1936 | **0.0000** | 1.0000 | 0.3607 | 0.1437 | **0.0244** | 0.4428 |
| D39 | **0.0000** | **0.0000** | **0.0000** | **0.0000** | **0.0029** | **0.0000** | 1.0000 | 1.0000 | **0.0000** | 1.0000 | **0.0440** | 0.1241 | **0.0000** | 0.5894 |
| D40 | **0.0000** | **0.0000** | **0.0000** | 1.0000 | **0.0411** | **0.0117** | 1.0000 | 0.5034 | **0.0000** | 0.7419 | 1.0000 | **0.0010** | **0.0000** | **0.0029** |
| D45 | 1.0000 | 0.5885 | 0.0606 | 1.0000 | 0.6999 | **0.0078** | **0.0362** | 1.0000 | 1.0000 | 0.0997 | 1.0000 | **0.0010** | **0.0000** | **0.0010** |
| D48 | 0.3695 | **0.0137** | **0.0029** | **0.0244** | 0.1603 | **0.0029** | 1.0000 | 0.3744 | 0.0587 | 1.0000 | 0.3392 | **0.0000** | 0.6999 | 0.1193 |
| D56 | 0.1066 | **0.0000** | **0.0010** | **0.0000** | 1.0000 | **0.0225** | 0.1173 | 0.2092 | 0.2551 | 1.0000 | 1.0000 | 0.2287 | **0.0049** | 1.0000 |
| D58 | 1.0000 | **0.0215** | 1.0000 | 0.6168 | **0.0010** | **0.0039** | **0.0020** | **0.0127** | 1.0000 | 1.0000 | 1.0000 | **0.0000** | **0.0000** | **0.0000** |
| D64 | **0.0000** | **0.0000** | **0.0000** | **0.0000** | 0.0753 | **0.0000** | **0.0000** | **0.0000** | **0.0000** | 1.0000 | 0.6676 | **0.0010** | **0.0000** | **0.0000** |
| D67 | 0.1114 | **0.0000** | **0.0254** | 0.0508 | **0.0049** | 0.0547 | 0.3353 | 0.7918 | **0.0000** | 0.2043 | 1.0000 | 0.6100 | 0.1466 | **0.0010** |
| D70 | **0.0000** | **0.0010** | **0.0000** | **0.0000** | 0.6432 | 1.0000 | **0.0000** | **0.0362** | **0.0000** | 0.2903 | 0.3920 | **0.0010** | **0.0000** | 1.0000 |
| D77 | 0.3548 | **0.0411** | 1.0000 | **0.0000** | 0.6061 | 0.3646 | **0.0010** | 0.1417 | 1.0000 | 1.0000 | 1.0000 | **0.0049** | 0.7234 | **0.0029** |
| D81 | **0.0000** | **0.0029** | **0.0000** | **0.0000** | **0.0000** | **0.0000** | **0.0000** | **0.0010** | **0.0362** | 0.7879 | 1.0000 | **0.0000** | **0.0000** | **0.0000** |
| D83 | **0.0196** | 1.0000 | 1.0000 | **0.0010** | 0.2209 | 0.1828 | 0.0518 | 1.0000 | **0.0254** | 1.0000 | 1.0000 | **0.0000** | **0.0029** | **0.0000** |
| D84 | 0.2502 | **0.0000** | **0.0000** | **0.0000** | **0.0000** | **0.0000** | 1.0000 | 1.0000 | **0.0000** | 0.4721 | 0.1505 | **0.0010** | **0.0000** | 0.0968 |
| D88 | 0.1887 | 0.0596 | **0.0352** | 1.0000 | 0.8143 | 0.1232 | 0.5797 | **0.0137** | 1.0000 | 0.7116 | **0.0391** | **0.0420** | 1.0000 | **0.0000** |
| D92 | 0.0968 | 0.2170 | **0.0000** | **0.0000** | 0.0792 | **0.0000** | 1.0000 | **0.0068** | **0.0176** | 1.0000 | 1.0000 | 0.1183 | **0.0000** | **0.0186** |
| D93 | 0.8045 | 1.0000 | 0.2903 | **0.0000** | 0.1369 | 0.2571 | 1.0000 | 1.0000 | 0.0899 | 1.0000 | **0.0196** | 0.7869 | 0.0880 | 0.0948 |
| D97 | 1.0000 | 0.4897 | **0.0264** | 0.0616 | 1.0000 | **0.0264** | **0.0029** | 0.1281 | 1.0000 | 1.0000 | 1.0000 | **0.0000** | **0.0010** | **0.0000** |
| D99 | 0.0890 | **0.0000** | **0.0000** | **0.0000** | **0.0000** | **0.0000** | **0.0381** | **0.0010** | **0.0000** | **0.0088** | **0.0088** | **0.0000** | **0.0000** | 0.1222 |
| D101 | 0.4027 | **0.0274** | **0.0029** | 0.4565 | 0.1730 | **0.0000** | 0.2346 | 0.3109 | **0.0000** | 1.0000 | 0.1769 | **0.0000** | 0.1202 | 0.0508 |
| D111 | **0.0000** | **0.0000** | **0.0000** | **0.0000** | **0.0000** | **0.0000** | **0.0000** | **0.0000** | **0.0000** | 0.7879 | 0.3969 | **0.0000** | **0.0000** | **0.0000** |
| D114 | **0.0029** | **0.0000** | **0.0000** | **0.0000** | **0.0000** | **0.0000** | **0.0000** | **0.0000** | **0.0000** | 1.0000 | 1.0000 | 0.4937 | **0.0450** | 0.0763 |
| D118 | **0.0000** | **0.0000** | **0.0000** | **0.0000** | **0.0000** | **0.0000** | **0.0000** | **0.0000** | **0.0000** | 0.1417 | **0.0010** | **0.0000** | **0.0000** | **0.0000** |
| D122 | **0.0059** | **0.0000** | **0.0000** | **0.0029** | **0.0020** | **0.0000** | 0.6755 | 0.0802 | **0.0000** | 1.0000 | 1.0000 | 1.0000 | **0.0000** | 1.0000 |
| D124 | **0.0264** | **0.0000** | **0.0000** | 0.1378 | **0.0342** | **0.0000** | **0.0010** | **0.0000** | **0.0000** | 0.0909 | **0.0000** | **0.0108** | 1.0000 | 0.7595 |
| D125 | 0.1593 | **0.0000** | **0.0000** | **0.0186** | **0.0000** | **0.0000** | 0.0508 | **0.0088** | **0.0029** | 0.4252 | 1.0000 | 0.7478 | 0.6012 | 0.7889 |
| D128 | 0.0802 | **0.0420** | **0.0000** | **0.0000** | 0.2424 | **0.0499** | **0.0029** | **0.0166** | **0.0196** | 1.0000 | 0.6432 | 0.6413 | **0.0020** | **0.0254** |
| D131 | 0.0577 | **0.0000** | **0.0000** | **0.0000** | **0.0000** | **0.0000** | 1.0000 | 0.1535 | **0.0020** | 1.0000 | 0.4340 | 0.1241 | **0.0000** | 0.1828 |
| D133 | 0.2366 | **0.0000** | **0.0000** | **0.0039** | 0.5347 | **0.0244** | 1.0000 | 0.3451 | **0.0000** | 1.0000 | 1.0000 | **0.0000** | **0.0000** | **0.0000** |
| D136 | 0.0792 | 0.4135 | 0.3636 | **0.0000** | 0.1535 | **0.0000** | **0.0342** | 0.1476 | **0.0088** | 1.0000 | 1.0000 | 1.0000 | 1.0000 | **0.0000** |

**Supplementary Table (Continued)**

| Loci | CentralSpanish | Uruguayan | ChihuahuaMexican | JaliscoMexican | MexicoMexican | VeracruzMexican | YucatanMexican | Mexican Amerindian | Tujia |
| --- | --- | --- | --- | --- | --- | --- | --- | --- | --- |
| D6 | 0.0675 | 1.0000 | **0.0000** | **0.0049** | 0.3578 | **0.0068** | **0.0000** | **0.0000** | **0.0000** |
| D39 | **0.0000** | **0.0000** | **0.0000** | **0.0000** | **0.0000** | **0.0000** | **0.0000** | **0.0000** | **0.0000** |
| D40 | **0.0000** | **0.0127** | 1.0000 | 1.0000 | 0.0596 | 0.1369 | **0.0049** | **0.0049** | **0.0000** |
| D45 | **0.0049** | **0.0000** | **0.0000** | **0.0000** | **0.0000** | **0.0000** | **0.0000** | **0.0000** | 1.0000 |
| D48 | **0.0372** | **0.0332** | **0.0313** | **0.0088** | **0.0000** | **0.0010** | **0.0000** | **0.0000** | 1.0000 |
| D56 | **0.0010** | 0.8407 | **0.0000** | **0.0000** | **0.0000** | **0.0000** | **0.0000** | **0.0000** | 0.2024 |
| D58 | **0.0010** | 0.2395 | **0.0000** | **0.0000** | **0.0000** | **0.0000** | **0.0000** | **0.0000** | 1.0000 |
| D64 | **0.0049** | 0.3089 | **0.0000** | **0.0000** | **0.0000** | **0.0000** | **0.0000** | **0.0000** | **0.0000** |
| D67 | 1.0000 | 0.1261 | **0.0000** | **0.0000** | **0.0000** | **0.0000** | **0.0000** | **0.0000** | **0.0000** |
| D70 | 1.0000 | 1.0000 | **0.0000** | **0.0000** | **0.0000** | **0.0000** | **0.0000** | **0.0000** | **0.0098** |
| D77 | 0.0557 | **0.0078** | 0.1633 | 0.1183 | **0.0039** | 1.0000 | 0.4809 | 0.1173 | 0.4976 |
| D81 | **0.0000** | **0.0000** | **0.0000** | **0.0000** | **0.0000** | **0.0000** | **0.0000** | **0.0000** | **0.0000** |
| D83 | **0.0000** | **0.0196** | **0.0000** | **0.0000** | **0.0000** | **0.0000** | **0.0000** | **0.0000** | 1.0000 |
| D84 | 0.2542 | **0.0010** | **0.0000** | 0.1222 | **0.0000** | 0.1603 | **0.0098** | **0.0010** | **0.0000** |
| D88 | **0.0049** | 0.3343 | 0.1486 | 0.1163 | **0.0000** | **0.0000** | **0.0000** | 0.1134 | 1.0000 |
| D92 | 0.4252 | **0.0000** | 1.0000 | 0.6041 | 0.8368 | 1.0000 | **0.0372** | 1.0000 | **0.0049** |
| D93 | 0.1515 | 1.0000 | **0.0000** | **0.0039** | 0.0538 | **0.0000** | **0.0000** | **0.0000** | 1.0000 |
| D97 | **0.0088** | **0.0000** | **0.0000** | **0.0000** | **0.0039** | **0.0020** | **0.0000** | **0.0000** | 0.1056 |
| D99 | **0.0108** | **0.0088** | **0.0000** | 0.1075 | **0.0000** | 1.0000 | **0.0010** | **0.0127** | **0.0000** |
| D101 | 1.0000 | 1.0000 | 0.2630 | 1.0000 | **0.0137** | 1.0000 | 1.0000 | 0.3412 | **0.0284** |
| D111 | **0.0000** | **0.0000** | **0.0000** | **0.0000** | **0.0000** | **0.0000** | **0.0000** | **0.0000** | **0.0000** |
| D114 | 0.0958 | 1.0000 | **0.0020** | **0.0225** | **0.0010** | **0.0000** | **0.0000** | **0.0029** | **0.0000** |
| D118 | **0.0000** | **0.0000** | 1.0000 | 0.9091 | 0.7537 | 0.6510 | 1.0000 | **0.0010** | **0.0000** |
| D122 | 0.5171 | 0.0626 | **0.0000** | **0.0000** | **0.0000** | **0.0000** | **0.0000** | **0.0000** | **0.0000** |
| D124 | 1.0000 | 1.0000 | **0.0000** | **0.0000** | **0.0000** | **0.0000** | **0.0000** | **0.0000** | **0.0088** |
| D125 | 0.4487 | 0.1740 | **0.0000** | **0.0020** | **0.0000** | **0.0000** | **0.0000** | **0.0000** | **0.0000** |
| D128 | 0.3060 | 0.1936 | **0.0020** | **0.0029** | **0.0000** | **0.0000** | **0.0000** | **0.0000** | 0.1154 |
| D131 | 0.4790 | **0.0293** | 1.0000 | 0.1310 | **0.0000** | **0.0000** | 0.0802 | **0.0000** | **0.0000** |
| D133 | **0.0000** | **0.0029** | **0.0088** | **0.0362** | **0.0010** | **0.0068** | **0.0000** | **0.0000** | **0.0108** |
| D136 | 0.2698 | 0.0655 | 0.1496 | **0.0176** | 0.5337 | 0.1799 | 0.4487 | **0.0000** | 0.4203 |
